# Targeted eDNA Metabarcoding Reveals New Populations of a Range‐Limited Stonefly

**DOI:** 10.1002/ece3.71244

**Published:** 2025-04-03

**Authors:** Graham A. McCulloch, Stephen R. Pohe, Shaun P. Wilkinson, Tom J. Drinan, Jonathan M. Waters

**Affiliations:** ^1^ Department of Zoology University of Otago Dunedin New Zealand; ^2^ Pohe Environmental Whangārei New Zealand; ^3^ Wilderlab NZ Ltd. Wellington New Zealand; ^4^ Department of Conservation Wellington New Zealand

**Keywords:** eDNA metabarcoding, Maungatua stonefly, Plecoptera, primer design

## Abstract

Understanding the geographic distributions of rare species can be crucial for conservation management. New environmental DNA (eDNA) technologies offer the potential to efficiently document the distributions of endangered species, but to date, such screening has focused largely on vertebrate taxa. Here we use freshwater eDNA to assess the geographic distribution of the Maungatua stonefly, *Zelandoperla maungatuaensis*, a flightless insect previously known from only a handful of streams draining a 4‐km section of the Maungatua mountain range in southern New Zealand. We analyzed freshwater eDNA from 12 stream localities across the Maungatua range. Screening with commercial eDNA COI primers failed to detect the focal species *Z. maungatuaensis*. However, newly designed species‐specific primers detected this taxon from four adjacent east‐flowing streams known to contain *Z. maungatuaensis*, and two streams from which it had not previously been detected. Subsequent manual surveys confirmed the presence of two newly discovered *Z. maungatuaensis* populations, with COI barcoding revealing that they together represent a previously unknown, genetically divergent subclade. Our results illustrate the potential of eDNA metabarcoding to help delineate the geographic ranges of rare taxa, and highlight the importance of primer specificity when screening for rare taxa. These findings also have considerable implications for commercial companies offering biodiversity and stream health eDNA services targeting invertebrates.

## Graphical abstract

We developed new species‐specific eDNA primers to detect *Zelandoperla maungatuaensis*, a rare, flightless insect restricted to a 4‐km stretch of the Maungatua range (South Island, New Zealand). eDNA metabarcoding confirmed that *Z. maungatuaensis* has a very narrow geographic range and revealed two previously unknown populations. Subsequent manual surveys validated the eDNA screening results, with additional COI sequencing revealing that these new populations form a distinct subclade.

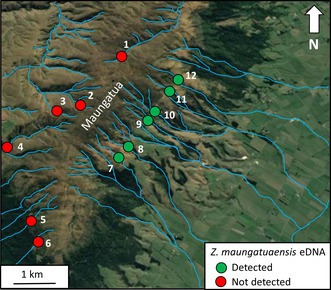

## 1 Introduction

Montane streams frequently house distinctive assemblages of unique headwater species (Clarke et al. 2008; Meyer et al. 2007; Richardson 2019). Many upland freshwater taxa have relatively restricted geographic ranges (e.g., Jordan et al. 2016; McCulloch, Dutoit, et al. 2022; Tsyrlin et al. 2021), reflecting their specialised habitat requirements and often limited dispersal abilities (Waters et al. 2020). These species are thus often particularly vulnerable to extinction (Giersch et al. 2015; Hotaling et al. 2017; Niedrist and Füreder 2023). Understanding the geographic distributions and diversity of such lineages is increasingly important in the context of anthropogenic threats to freshwater ecosystems, including climate change (Birrell et al. 2020; Hotaling et al. 2017; Khamis et al. 2014) and the spread of invasive species (Haag 2019).

Monitoring headwater stream biodiversity can often be challenging. Accessing remote upland streams can be difficult, and the small invertebrate species that often dominate such systems can be challenging to sample and identify without specialized fieldwork skills and taxonomic expertise. Recently developed environmental DNA (eDNA) metabarcoding approaches provide a promising toolbox addition to traditional freshwater biodiversity surveys (Thomson et al. 2012). These methods require less taxonomic expertise, and DNA can potentially be detected at significant distances downstream from target invertebrate populations (Deiner and Altermatt 2014). These eDNA approaches have been used to detect a range of rare freshwater vertebrate taxa (e.g., McColl‐Gausden et al. 2023; Thomson et al. 2012; Tingley et al. 2021), and are commonly used to screen for invertebrate taxa (Fan et al. 2025; Macher et al. 2016; Waters et al. 2024; Wilkinson et al. 2024), with several recent studies suggesting that these approaches may have the potential to detect relatively small or rare invertebrates (Doi et al. 2017; Tsyrlin et al. 2021).

**FIGURE 1:**
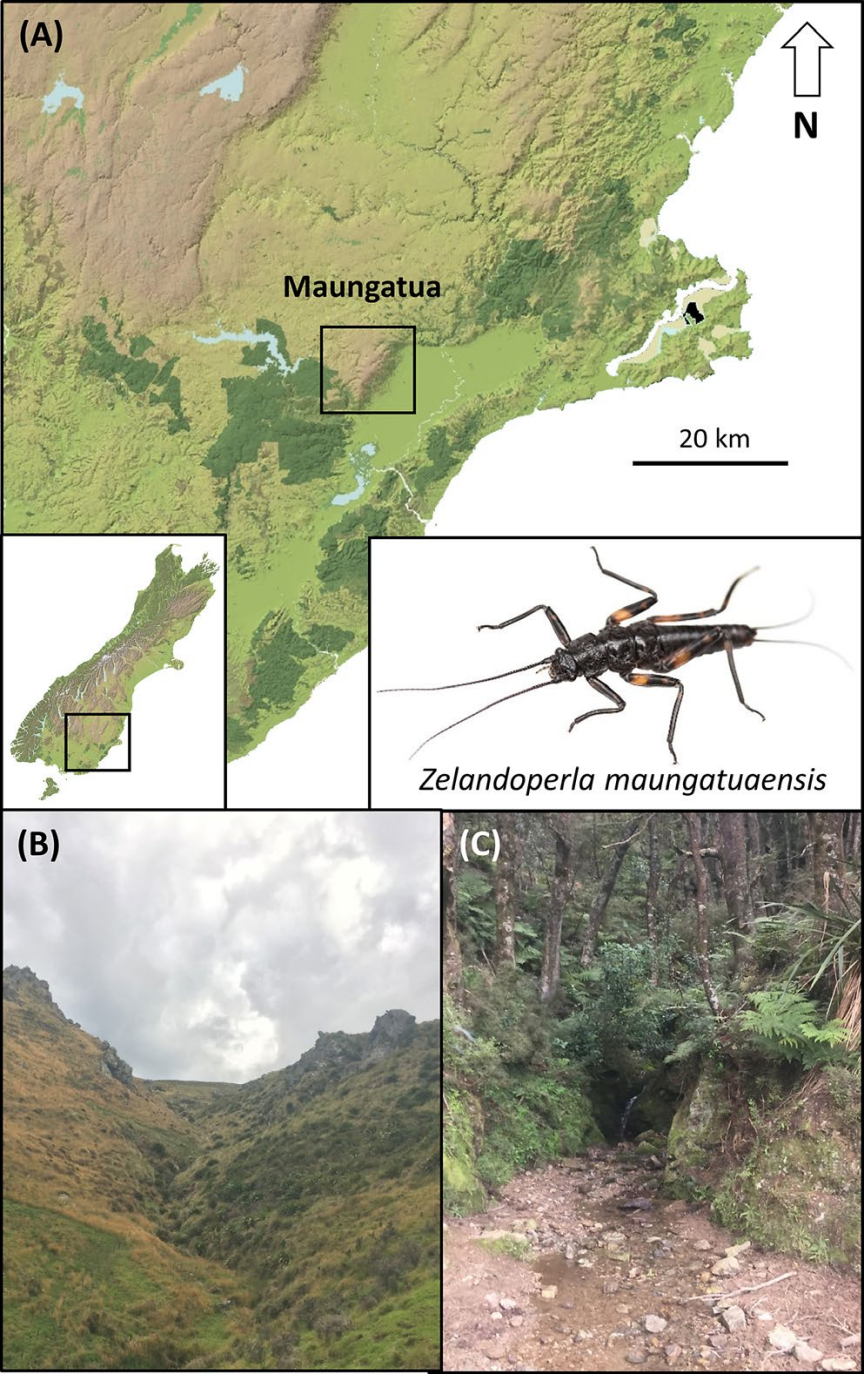
The endemic flightless stonefly *Zelandoperla maungatuaensis* has a highly restricted distribution on the isolated Maungatua range (A) in southeastern South Island, New Zealand. This range has an elevation of 895 m. Light brown = upland regions, green = forest, light blue = lakes. This species is restricted to a handful of small streams (B, C) draining the eastern flanks of this range.

In this study, we use eDNA metabarcoding to reassess the geographic distribution and genetic diversity of the enigmatic Maungatua stonefly, *Zelandoperla maungatuaensis* Foster. This recently discovered flightless stonefly is thought to have an extremely narrow geographic range, having been recorded only from a 4‐km stretch of the isolated Maungatua mountain range in southeast New Zealand (Figure 1A; Foster et al. 2020; McCulloch, Foster, et al. 2022). Intriguingly, despite its apparently narrow geographic range, this species boasts exceptionally high mitochondrial and genome‐wide diversity, with three divergent subclades detected (McCulloch, Foster, et al. 2022). This species is unusual for a mainland species in having such a localised distribution (especially for such a divergent lineage) in the absence of any obvious physical barriers (McCulloch, Foster, et al. 2022). Such a localised distribution is more typical of oceanic island endemic taxa (Vangestel et al. 2024). Ecologically, this insect species is distinctive globally in being wing‐reduced and flightless, as the vast majority of plecopteran taxa are flighted (Fochetti and De Figueroa 2008).

Preliminary surveys located *Z. maungatuaensis* from only a handful of streams, typically above the alpine treeline (Foster et al. 2020)—habitat types that are frequently dominated by wing‐reduced freshwater insects (McCulloch, Foster, Ingram, and Waters 2019). However, the heavily incised streams draining the steep upper slopes of this mountain range (Figure 1B,C) are particularly difficult to access, so the full range of the species remains unknown. In this study, we use eDNA metabarcoding to reassess the geographic distribution of this enigmatic, narrow‐range upland species. Specifically, we undertake eDNA metabarcoding analyses of water samples collected from 12 localities across the Maungatua range, develop novel metabarcoding primers to detect *Z. maungatuaensis*, and conduct manual surveys to confirm the eDNA results. Our study demonstrates the potential of eDNA approaches for detecting rare freshwater invertebrates and for informing the conservation of range‐restricted species.

## 2 Materials and Methods

### 2.1 eDNA Sampling

**FIGURE 2:**
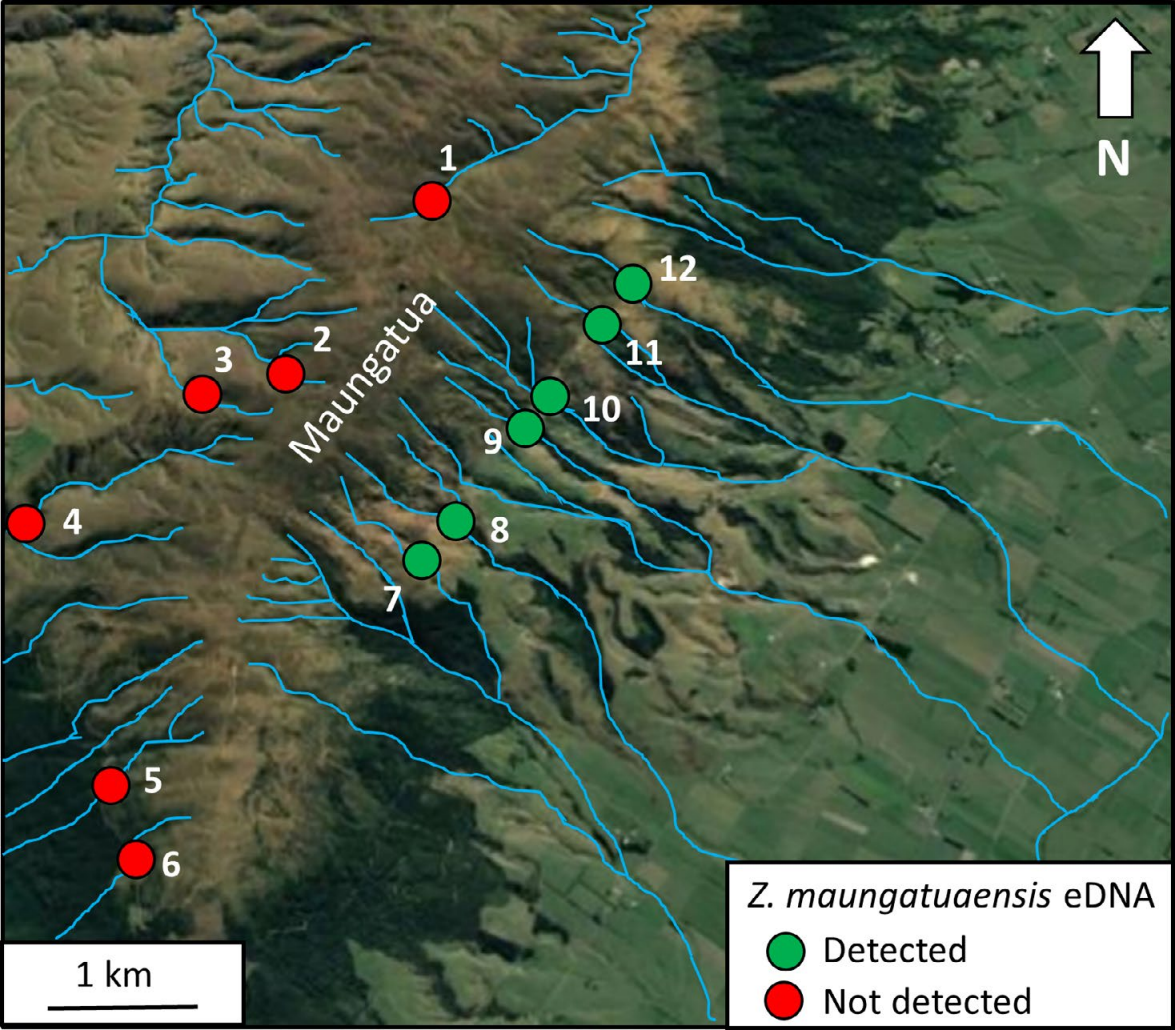
Freshwater environmental DNA sampling localities on the Maungatua range (see Table 1 for further details). *Zelandoperla maungatuaensis* eDNA was detected in all samples from streams draining the eastern slopes of the Maungatua range (green circles), but not from any streams draining the northern, western, or southern flanks (red circles).

*Zelandoperla maungatuaensis* lives in and around narrow, high‐gradient stream habitats, where it spends most of its life as a freshwater nymph, with only a short‐lived terrestrial adult phase (Foster et al. 2020). We collected water samples from 12 narrow (< 1 m wide) streams across the Maungatua range in early March 2023 (Figure 2; Table 1). The sampling design incorporated four ‘positive controls’, i.e. streams draining the eastern side of the range from which *Z. maungatuaensis* had been previously recorded (streams 7–10; Table 1). We also collected water samples from two further eastern sites that had not previously been surveyed (streams 11–12). Additionally, we collected samples from six neighbouring (south‐, west‐ and north‐draining) streams outside the known range of this species. Water was filtered onto a Wilderlab 1.2‐μm cellulose acetate eDNA column using a 50‐ml syringe, and preserved immediately in DNA/RNA Shield (Zymo). Five 1‐l subsamples of stream water were filtered per stream, a total of 60 subsamples. Subsamples were taken from different mesohabitats (e.g., fast‐flowing rapid, pools) within a 20‐m stretch of stream to maximise the probability of detecting *Z. maungatuaensis*.

**TABLE 1:** Details of water sampling sites included in environmental DNA analyses; numbers of subsamples per site in which *Zelandoperla maungatuaensis* DNA was detected (with newly designed species‐specific primers), and mean numbers of *Z. maungatuaensis* reads per site.

Site code	Northing	Easting	Collection date	Elevation	*Z. maungatuaensis* eDNA	
					Detections	Mean no. of reads (range)
1	−45.87252	170.11865	8/03/2023	840	0/5	0
2	−45.88307	170.10708	1/03/2023	840	0/5	0
3	−45.88365	170.10070	1/03/2023	780	0/5	0
4	−45.89075	170.08405	1/03/2023	550	0/5	0
5	−45.89778	170.08560	1/03/2023	560	0/5	0
6	−45.90138	170.08905	1/03/2023	540	0/5	0
7	−45.89393	170.11635	8/03/2023	570	5/5	1438 (883–1670)
8	−45.89203	170.11798	8/03/2023	560	3/5	845 (0–3158)
9	−45.88613	170.12523	8/03/2023	570	5/5	2155 (28–5512)
10	−45.88523	170.12688	8/03/2023	580	4/5	3643 (0–10,690)
11	−45.88080	170.13248	8/03/2023	620	5/5	1564 (6–3136)
12	−45.87912	170.13560	8/03/2023	590	2/5	129 (0–287)

### 2.2 Primer Design

Prior to sample processing, we conducted *in silico* analyses to determine whether conventional COI primers used in the Wilderlab ‘basic freshwater panel’ (Table S1; Wilkinson et al. 2024) were likely to amplify *Z. maungatuaensis* eDNA. The conventional COI primers in this panel amplify a 76‐bp amplicon (Table 2). Two mismatches were detected at the forward primer binding site, and another five mismatches were detected in the reverse primer binding site (Figure S1). As these mismatches could potentially prevent primer binding, we designed a novel primer combination to amplify the same 76‐bp fragment (Table 2).

**TABLE 2:** Details of the conventional Wilderlab COI primers and newly designed *Zelandoperla maungatuaensis*‐specific COI primers used in this study. Both primer combinations have an annealing temperature of 45°C and amplify a 76‐bp fragment.

Assay	Forward primer	Reverse primer	Reference
Conventional	DACWGGWTGAACWGTWTAYCCHCC	GTTGTAATAAAATTAAYDGCYCCTARAATDGA	Leray et al. 2013; Vamos et al. 2017
Species‐specific	DACWGGNTGAACWGTNTAYCCHCC	AATAAAGTTCACTGCCCCCAAGATTGA	Wilderlab in house

### 2.3 Sample Processing

Preserved eDNA samples were processed by Wilderlab (https://www.wilderlab.co.nz/) using both conventional and newly designed *Z. maungatuaensis*‐specific primer combinations. This approach allowed us to determine whether *Z. maungatuaensis* could be detected with the conventional primers and also to assess insect diversity within the streams (which would not be possible with the newly designed species‐specific primers). DNA extraction and PCR were conducted in a sterile, compartmentalised laboratory, following the protocols of Wilkinson et al. (2024). Sequencing was done on an Illumina iSeq 100 system at Wilderlab (Wellington, New Zealand). Primer sequences and annealing temperatures are provided in Table S1. An internal negative control was included on each of the two sequencing runs denoted WL0375 and WL0535, using nuclease‐free water (IDT, Singapore) in place of the extracted DNA template. Demultiplexed sequences were quality‐filtered with a de‐novo chimera removal step to produce amplicon sequence variants (ASVs) using DADA2 (Callahan et al. 2016) assigned to respective taxa based on the NCBI GenBank database (https://www.ncbi.nlm.nih.gov/) using an exact‐match search, followed by the SINTAX classification method (Edgar 2016; with the sensitivity threshold set to 0.99) for any unmatched ASVs.

### 2.4 Manual Surveys and Phylogenetic Analyses

Manual sampling of invertebrate populations was subsequently undertaken in the two streams where previously unknown populations of *Z. maungatuaensis* were detected by eDNA, with permission from the New Zealand Department of Conservation. To examine the phylogenetic relationships of the newly detected *Z. maungatuaensis* specimens, we sequenced a 640‐bp portion of the mitochondrial COI gene of three individuals from each stream (following the approach of McCulloch, Foster, et al. 2022).

The six newly amplified COI sequences were aligned with 36 *Z. maungatuaensis* sequences downloaded from GenBank, and the phylogenetic relationships of these sequences were assessed using a Bayesian approach, implemented in Mr. Bayes 3.2.7a (Ronquist et al. 2012). *Zelandoperla agnetis* McLellan (GQ414594) and *Zelandoperla denticulata* McLellan (GQ414593) sequences were included as outgroups. We ran four Markov chains for 3 million generations, with chains sampled every 200 generations. The first 2000 trees were discarded as burn‐in. We used Tracer v1.7.0 (Rambaut et al. 2018) to confirm that all of the parameters had converged and to ensure that the effective sample size was greater than 200 for each of the priors.

## 3 Results

Initial screening of water samples with the conventional Wilderlab eDNA primers detected 39 EPT taxa (14 Ephemeroptera, 12 Plecoptera, 13 Trichoptera) across the 12 streams (60 eDNA tests). The south‐draining (sites 4–6) and east‐draining streams (sites 8–12) recorded 7–20 EPT taxa per stream (mean = 13.7; Figure S2). By contrast, the two north‐draining streams (sites 2 and 3) contained fewer taxa (mean 5.5 EPT taxa per stream; Figure S2). Conventional Wilderlab eDNA primers, however, did not detect *Z. maungatuaensis* DNA in any of the 12 streams sampled (0/60 eDNA tests).

By contrast, species‐specific eDNA metabarcoding analysis detected *Z. maungatuaensis* DNA in all six east‐draining streams along the Maungatua range, including two streams from which *Z. maungatuaensis* had not previously been recorded (streams 10 and 11; Figure 2a; Table 1). In total, *Z. maungatuaensis* eDNA was detected in 24/30 (80%) subsamples across the six east‐draining streams (Table 1). For the six east‐draining streams, detection rates within individual streams ranged from 40% (site 12) to 100% (sites 7, 9, and 11), with substantial variation in the number of reads per subsample (Table 1). In addition, five of these streams (sites 7–10, and 12) also yielded DNA of the congeneric *Zelandoperla fenestrata* Tillyard, implying that the two species co‐exist. By contrast, neither *Z. maungatuaensis* eDNA nor *Z. fenestrata* eDNA was detected in any of the flanking streams draining the northern, western, or southern slopes of the Maungatua range.

**FIGURE 3:**
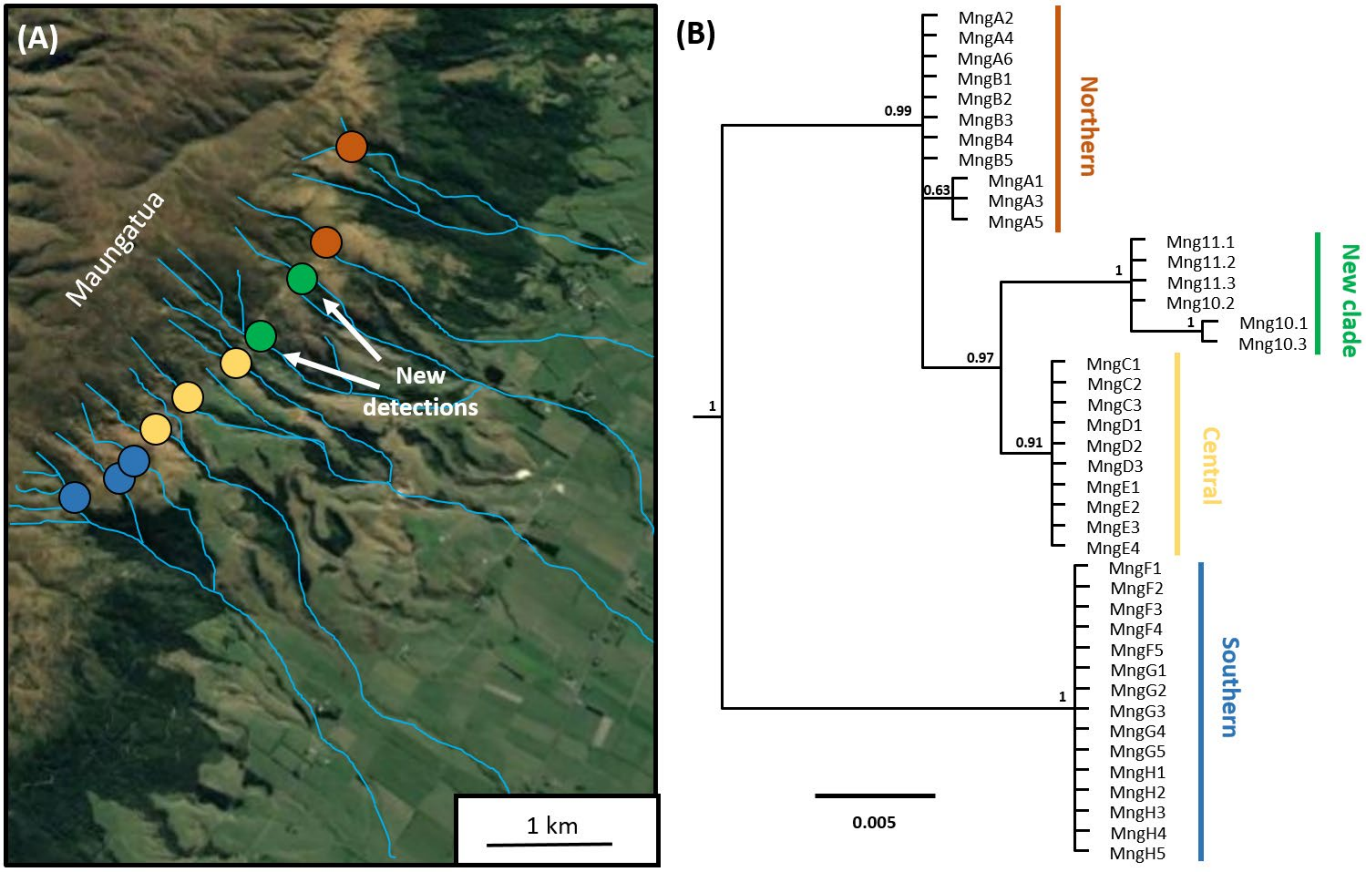
A newly‐detected subclade of *Zelandoperla maungatuaensis*. (A) Records of *Z. maungatuaensis*, coloured by COI clade (see McCulloch, Dutoit, et al. 2022), with the newly‐detected linage indicated in green. (B) Bayesian phylogeny of mitochondrial COI sequences, illustrating the relationships among *Z. maungatuaensis* lineages (sample codes based on McCulloch, Foster, et al. 2022; GenBank Accession numbers OM802728‐OM802761). Posterior probabilities are noted above each node. Outgroups (*Zelandoperla agnetis* and *Zelandoperla denticulata*) are excluded for diagrammatic clarity.

Subsequent manual surveys located novel populations of *Z. maungatuaensis* nymphs in streams 10 and 11, validating eDNA results from these newly‐discovered stream populations. Mitochondrial COI sequencing of these samples revealed two unique *Z. maungatuaensis* haplotypes, with phylogenetic analysis indicating that these samples together form a novel subclade that is 1.3% divergent from the previously described “Central” *Z. maungatuaensis* clade (Figure 3).

## 4 Discussion

Our eDNA metabarcoding study from the Maungatua range revealed new populations of *Z. maungatuaensis*, and, with additional COI sequencing, previously undetected phylogenetic diversity within this highly range‐restricted stonefly. Furthermore, the absence of eDNA of this taxon from adjacent drainage systems further highlights the extremely narrow geographic range of this taxon (McCulloch, Foster, et al. 2022). Together, these findings highlight the utility of eDNA approaches for studies of freshwater biodiversity (Waters et al. 2024) and conservation biology (Sahu et al. 2023; Thomson et al. 2012), but also highlight the importance of primer specificity when screening for rare taxa.

### 4.1 Primer Design

Environmental DNA is becoming an increasingly powerful tool as researchers seek to discover the distributions and diversity of rare and/or endangered freshwater species (Sakai et al. 2019; Xia et al. 2021). However, ‘false negative’ results (where eDNA metabarcoding does not identify the taxon even though it is present in the sample) have the potential to severely hamper or misdirect conservation and monitoring efforts. These ‘false negatives’ may be due to technical issues with sample collection or processing, or can reflect limitations with the reference database (Ficetola et al. 2015). The results of the current study also emphasise that *in silico* analysis and primer design (Farrington et al. 2015; Wilcox et al. 2013) are crucial for detecting eDNA of some species for which ‘universal’ barcoding primers may lack specificity due to sequence mismatches (Ficetola et al. 2015). Specifically, we encourage researchers to ensure primer specificity when focussing on species that are rare, or have restricted distributions.

‘False negative’ results highlight a potential limitation of eDNA approaches for species discovery: to detect a particular species can require prior knowledge of its potential presence, and the development of specific primers needed to detect it. For example, New Zealand's Wilderlab public database (https://www.wilderlab.co.nz/explore) contains the results of more than four thousand eDNA surveys across the country, and none of these samples has previously yielded sequences of *Z. maungatuaensis*. Based on these eDNA data alone (that have relied on generic, non‐specific primers) it is presently impossible to rule out a wider distribution for this rare taxon; however unlikely that may be based on data from traditional invertebrate survey techniques.

### 4.2 Headwater Streams

Headwater lineages and other upland species frequently exhibit distinctive adaptations to their high elevation habitats (e.g., adaptation to local geological substrata; Hodkinson 2005; Jordan et al. 2016; McCulloch et al. 2021; Stokes et al. 2023), and such species often show strong local endemism (e.g., Kim et al. 2023; Stokes et al. 2023). Our detection of a novel, fourth subclade of *Z. maungatuaensis* within the narrow distribution of this extremely range‐limited taxon highlights the potential for even adjacent upland headwater systems to harbour unique freshwater lineages (Wishart and Hughes 2003). Indeed, recent studies suggest that both physical isolation (Waters et al. 2020) and local adaptation (Stokes et al. 2023) in headwater systems can play major roles in the generation of freshwater biodiversity (McCulloch, Foster, Dutoit, et al. 2019; McCulloch, Dutoit, et al. 2022; Penaluna et al. 2023; Suzuki et al. 2019). In the current study, the ancient genetic divergence between proximate populations of this non‐dispersive upland stonefly species implies a lack of historical connectivity among parallel streams. The deep genetic structure among stream populations, also evident for genome‐wide markers (McCulloch, Foster, et al. 2022), similarly suggests that these headwater populations have experienced relatively stable histories.

In summary, our study highlights the value of eDNA approaches for detecting new populations of rare freshwater species, resolving their biogeographic distributions, and revealing biodiversity in habitats that are challenging to sample. The use of such novel approaches offers great opportunities for conservation practitioners. In particular, this study demonstrates that targeted eDNA metabarcoding can be a valuable and cost‐effective tool to help target much‐needed conservation efforts for rare or endangered freshwater invertebrate species.

## Author Contributions

**Graham A. McCulloch:** conceptualization (equal), data curation (equal), formal analysis (equal), funding acquisition (equal), investigation (equal), project administration (equal), visualization (equal), writing – original draft (equal), writing – review and editing (equal). **Stephen R. Pohe:** conceptualization (equal), funding acquisition (equal), writing – review and editing (equal). **Shaun P. Wilkinson:** conceptualization (equal), data curation (equal), funding acquisition (equal), investigation (equal), methodology (equal), writing – original draft (equal). **Tom J. Drinan:** conceptualization (equal), funding acquisition (equal), writing – review and editing (equal). **Jonathan M. Waters:** conceptualization (equal), investigation (equal), writing – original draft (equal), writing – review and editing (equal).

## Conflicts of Interest

S.P.W. owns and operates Wilderlab NZ. Ltd., a commercial eDNA processing laboratory.

## Supporting information


Data S1.

## Data Availability

New COI barcode sequences generated in this study are available on GenBank (PV324758‐PV324763), and new metabarcoding sequences are available via the NCBI Sequence Read Archive (PRJNA1235809).
