# Supplementary material for: Targeted eDNA Metabarcoding Reveals New Populations of a Range‐Limited Stonefly
Source: Ecol Evol. 2025 Apr 3;15(4):e71244. doi: 10.1002/ece3.71244 (PMC11968413; doi:10.1002/ece3.71244)
Supplement: Supplementary file 1 — Data S1. [file ECE3-15-e71244-s001.docx]

**Supplementary Figures**

**
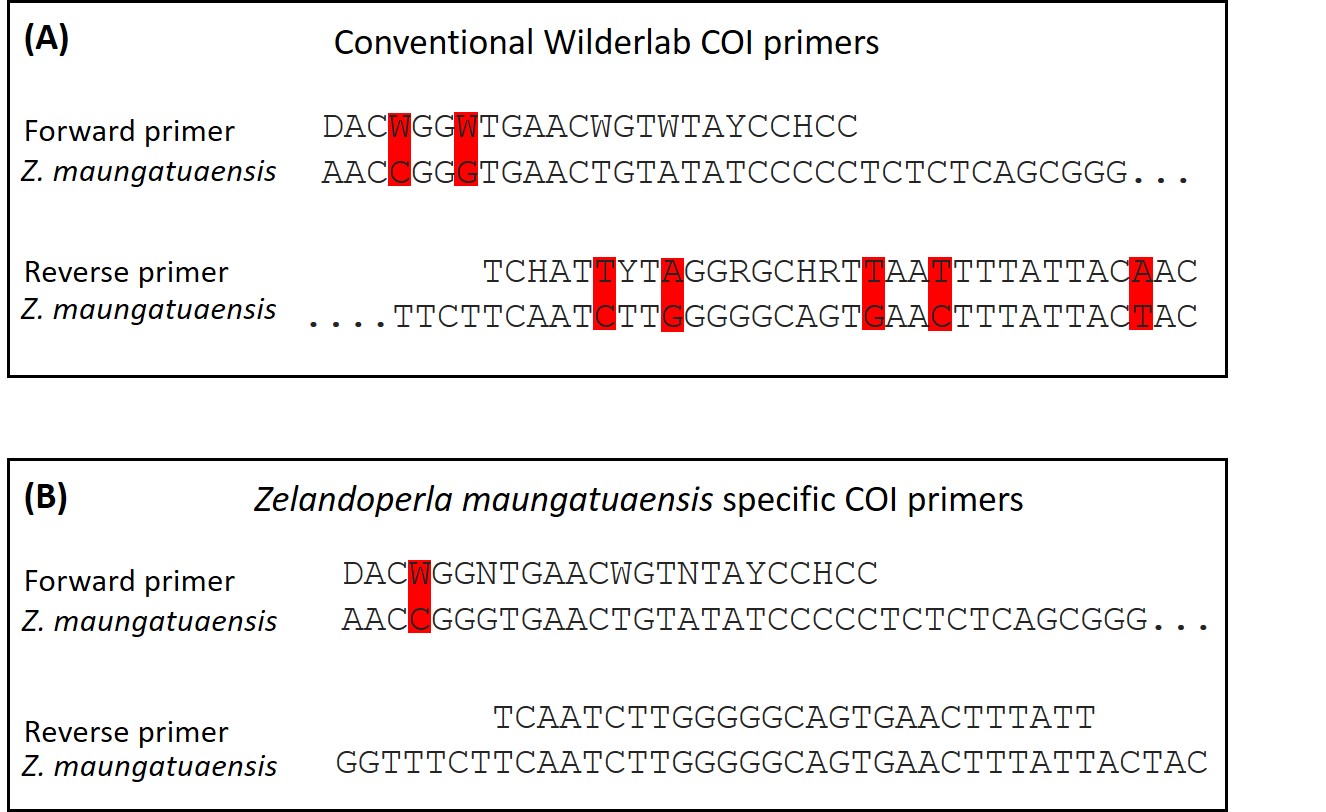
**

**Figure S1.** (A) An alignment of conventional Wilderlab COI primers with *Zelandoperla* *maungatuaensis* DNA, illustrating the high number of mismatches (highlighted in red) between the primers and the DNA sequence. (B) An alignment of the newly designed species-specific primers with *Z. maungatuaensis* DNA. Note that the newly designed forward primer was developed as a general insect metabarcoding primer, as opposed to being specific for *Z. maungatuaensis* (as was the intention for the new reverse primer). The degenerate base ‘W’ at position 4 in the new forward primer does not appear to inhibit binding for *Z. maungatuaensis*, despite being non-complementary to the Cytosine at this position.


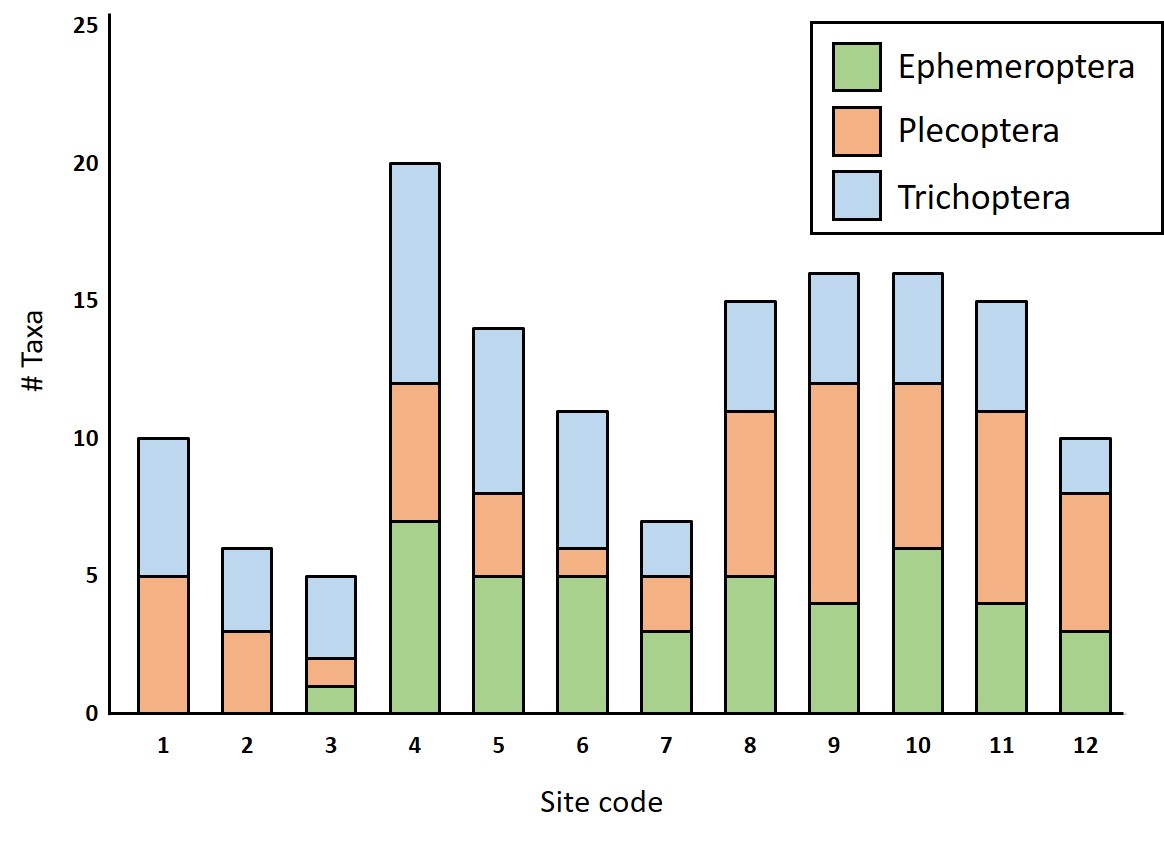


**Figure S2.** The number of EPT taxa identified from 12 streams along the Maungatua range using eDNA metabarcoding. See Table 1 for site details.

| **Table S1.** Mitochondrial primers used for eDNA metabarcoding. | | | | |  |  |  |  |  |
| --- | --- | --- | --- | --- | --- | --- | --- | --- | --- |
| Assay code | Target group | Gene region | Amplicon length | Forward primer length | Forward primer sequence | Reverse primer length | Reverse primer sequence | Annealing temp | Reference |
| LG | Fish | mt12S | 110-115 | 20 | CGGCGTAAAGWGTGGTTAGG | 27 | CATAGTGGGGTATCTAATCCCAGTTTG | 58 | Wilkinson (2023) https://s3.ap-southeast-2.amazonaws.com/wilderlab.resources/methods/Wilderlab_metabarcoding_methods_2.1.0.pdf |
| RV | Vertebrates | mt12S | 97-103 | 18 | TTAGATACCCCACTATGC | 18 | TAGAACAGGCTCCTCTAG | 58 | Riaz et al. (2011) https://doi.org/10.1093/nar/gkr732 |
| WV | Vertebrates | mt16S | 40-100 | 21 | GACGAGAAGACCCTWTGGAGC | 16 | CCRYGGTCGCCCCAAC | 58 | Adapted from Nester et al. (2020) https://doi.org/10.1002/edn3.93 |
| CI | Insects | COI | 78 | 24 | DACWGGWTGAACWGTWTAYCCHCC | 32 | GTTGTAATAAAATTAAYDGCYCCTARAATDGA | 45 | Wilkinson et al. (2024) https://peerj.com/articles/16963/ |
| XZ | Zelandoperla | COI | 78 | 21 | DACWGGNTGAACWGTNTAYCCHCC | 27 | AATAAAGTTCACTGCCCCCAAGATTGA | 45 | This study |
